# Stretching Scarce Authorizing Legislation as Far as Possible: A Legislative History of the 340B Drug Pricing Program

**DOI:** 10.1111/1468-0009.70090

**Published:** 2026-05-20

**Authors:** SAYEH NIKPAY, MIKAYLA REINKE, NICOLE QUINONES

**Affiliations:** ^1^ University of Minnesota School of Public Health

**Keywords:** 340B, drugs, safety‐net

## Abstract

**Policy Points**

- The original purpose of the 340B program was to exempt Public Health Service Act funded clinics and state and local public hospitals from the inflationary best‐price component of the recently enacted Medicaid drug rebate program.
- The secondary purpose was to reduce drug prices for these clinics and hospitals in order to preserve and expand access to free and discounted care.
- The cutoff for the disproportionate share hospital‐based eligibility criteria was selected as part of political compromises to qualify specific nonprofit hospitals.

**Context:**

The 1992 340B Drug Pricing Program (“340B”) started as a narrowly focused program aimed at Public Health Service Act–funded clinics and public hospitals. Today 340B includes two‐thirds of all nonprofit hospitals in the United States and accounts for more than $80 billion in discounted drug purchases. Statutory language authorizing 340B is sparse, and attempts to strengthen or cut the program have been stymied by lack of clarity on Congress's original intentions. The object of this study was to clarify Congress's original intentions for 340B.

**Methods:**

Our qualitative analysis was informed by the collection and analysis of two sources: (1) 175 internal primary source documents and (2) 19 structured interviews conducted with 18 key informants with respect to the creation of 340B.

**Findings:**

Congress had two intentions in establishing 340B in 1992. The first was to address an unintended consequence of the Medicaid Drug Rebate Program (MDRP), which raised costs on safety‐net clinics that received significant discounts on drugs prior to the rebate program. Such core safety‐net clinics operate on fixed budgets heavily dependent on federal grants, which also require them to provide free and discounted care to patients regardless of their ability to pay. The purpose of 340B was to exempt these clinics from the best price provision of the MDRP. The second was to establish minimum discounts for drug prices for core safety‐net providers. While public hospitals were added because of their safety‐net mission, the disproportionate share hospital eligibility criterion were included to qualify two specific hospitals to secure bipartisan support.

**Conclusions:**

Congress's original intention for 340B was to enable core safety‐net providers to continue to provide drugs to patients regardless of costs in the face of historic drug price increases set off by the MDRP. The current scope of the program exceeds Congress's original intent.

## Introduction

On june 4, 2024, the house energy and commerce committee held a hearing on the oversight of the 340B Drug Pricing Program (“340B”), which has entitled certain clinics and hospitals to discounts on outpatient drugs since 1992.^1^ The committee was deliberating over whether 340B was achieving Congress's intended goals. On one hand, Representative Kathy Castor (D‐FL) argued that “the 340B drug pricing program provides real savings to millions of Americans and helps hospital[s] and clinics extend care to patients that need it most.”^2^ On the other hand, committee co‐chair Cathy Rodgers (R‐WA) worried “that [340B] is being co‐opted by larger, more profitable health care systems that are using it solely for financial gain at the expense of the patients who [need] it [the] most.”^3^ Another member of the committee, committee co‐chair Morgan Griffith (R‐VA), alleged that “this is the reverse Robin Hood. Steal from the poor to pay the rich.”^4^

Underlying these contrasting assessments are two important, yet unanswered questions: Who was 340B designed to help, and how was it supposed to have helped them? This hearing, like others on the same topic, featured language from both supporters and critics of 340B about the “intentions of Congress,” and the “goals of the program” to allow participants to “stretch scarce Federal resources as far as possible, reaching more eligible patients and providing more services.” However, this language, often cited as Congress's original intention for the program, does not appear anywhere in the statute. The authorizing legislation for 340B simply defines the discounts, outlines two prohibitions on use of the drugs, and defines the parties that can participate in the program.^5^ As a result, very little of Congress's original intentions for 340B can be inferred by the statute. To uncover Congress's original intentions, one must consider the history—as assessed through draft legislation; hearing testimony; internal memos; and the statements of legislators, staff, lobbyists, and advocates who participated in the process of conceptualizing and proposing policy; and by navigating the politics of the ultimate passage of 340B's authorizing legislation, the Veterans Health Care Act of 1992.

To infer Congress's original intent for 340B, we collected and analyzed (1) more than 175 primary source policy documents from 1990–1992, and 19 structured interviews with 18 key informants who were directly involved in the creation and passage of 340B.^6^ These key informants included congressional staffers from both parties, employees from key executive branch offices in the George H.W. Bush administration, lawmakers, advocates representing health care organizations that eventually participated in the program, lobbyists and government relations employees for pharmaceutical manufacturers, and academic experts. (A detailed description of the methods is provided in the appendix.)

### Qualitative Analysis Reveals Original Legislative Intentions

We identified two main intentions of Congress when it established 340B. First, 340B was conceived and designed as a rapid fix to address unintended consequences of the Medicaid Drug Rebate Program (MDRP) in its first years of operation. The MDRP entitled state Medicaid agencies to the larger of a percentage discount off the average prices paid by nonfederal payers or the “best price” in the market. Although the rebate program lowered costs for the Medicaid program, the best prices available in the market were frequently those charged to “core safety‐net providers”: health care organizations with a mandate to provide care to patients regardless of ability to pay. This group included clinics funded by the Public Health Service Act (PHSA) and the Department of Veterans Affairs (VA). Although the discounts they had received before the MDRP were large, the volume of drugs purchased by these providers was relatively small, amounting to less than 0.1% of total drug spending,^7^ and thus represented a paltry amount of lost revenue for drug manufacturers. However, after the best price provision went into effect, manufacturers began to raise prices on both the PHSA clinics and the VA to avoid extending deeply discounted pricing to the entire Medicaid program, or 10% of the market. The Veterans Health Care Act of 1992 exempted these parties from the best price provision, achieving the short‐term objective of de‐linking core safety‐net provider prices from the prices paid by Medicaid agencies.

A second and longer‐term objective was to reduce drug prices for core safety‐net providers dependent on discretionary federal spending. Although they served different populations and had different levels of political support, both the PHSA clinics and the VA faced this problem. As drug prices began to rise after the MDRP, the share of these providers’ largely fixed budgets spent on drugs also rose. Both providers and policymakers feared that the newly squeezed budgets would jeopardize access to services (drug and nondrug alike) for patients who relied on the health care safety net. Although not originally included in initial proposals of what would become 340B, another core safety‐net provider—public hospitals—was added to later proposals of the program because they faced similar fixed budgets dependent on state and local government funds. Our analysis also revealed that the eventual dominance of nonprofit hospitals—which have neither public hospitals’ mandate to provide free or discounted care nor budgets depending on federal, state, or local grant revenue—was not the intention of policymakers.

In the following sections, we provide a summary of the 340B Drug Pricing Program, present the results of a legislative historical analysis based on the review of primary source documents and key informant interviews, and discuss our findings in the context of 340B today. A detailed description of the methods is provided in the appendix. In accordance with mutual agreements with the interviewees, we do not directly attribute quotes to specific named interviewees. In addition, some primary source documents analyzed are not publicly available. We therefore refer narratively to interviews and primary source documents in the text rather than with full citations. A list of interviewees (Table A1) and an inventory of nonpublic documents (Table A2) are provided in the appendix.


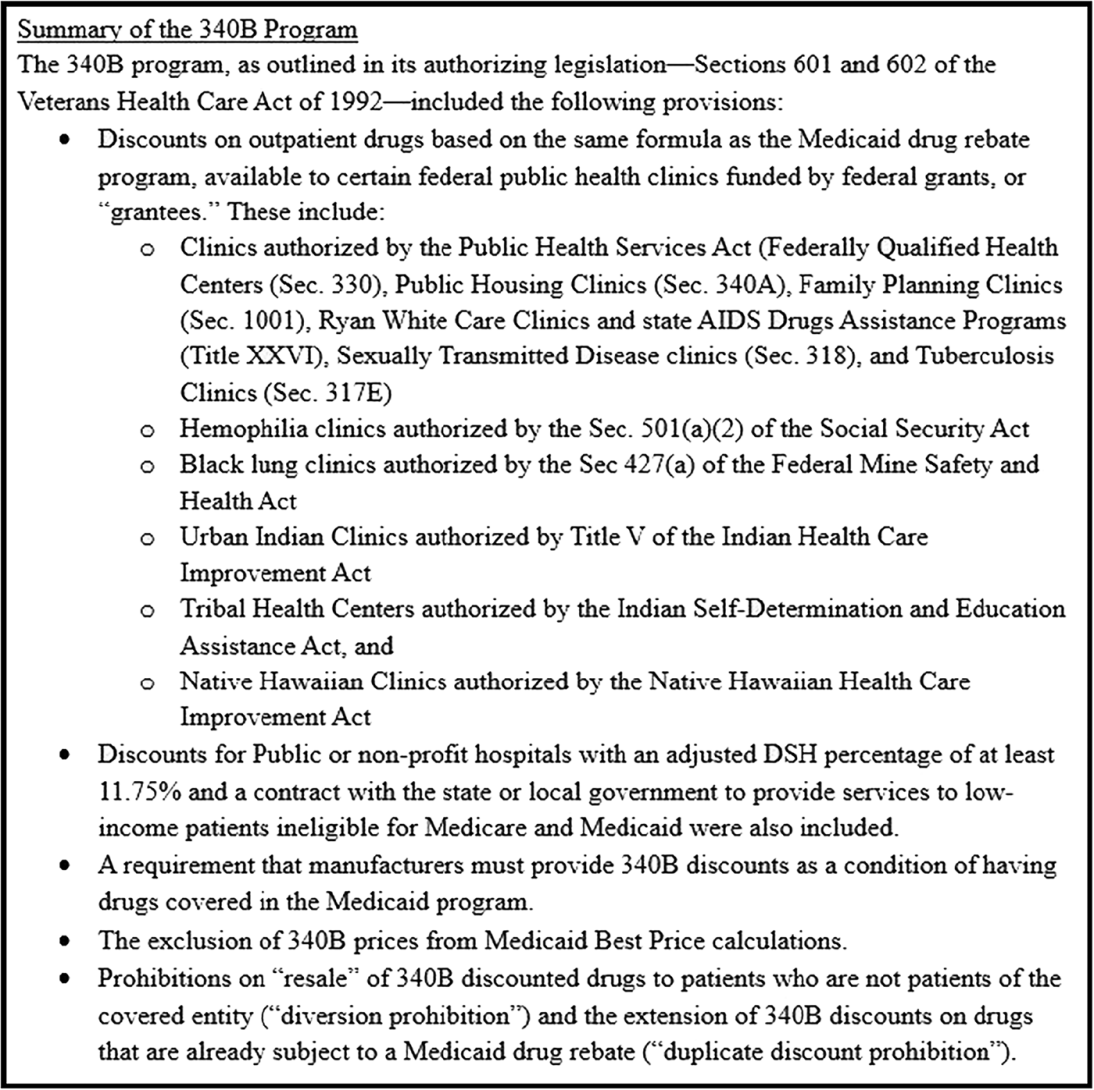


## Results

### Origins: The Medicaid Drug Rebate Program and Fallout From the Best Price Provision

Prescription drug prices were rising through the 1980s, creating affordability problems for Americans and the safety‐net programs that purchased drugs on their behalf. At this time, roughly 70% of drug spending was out‐of‐pocket, 20% was paid through private insurance, and 10% was paid through Medicaid.^8^ For high‐volume, private payers, such as commercial insurers and hospitals, manufacturers were thought to have granted discounts of between 40% and 70% off average wholesale prices—a kind of sticker price never paid by these high‐volume purchasers.^9^ Unlike these private purchasers, there was no mechanism for states or the federal government to negotiate drug prices. As a result, Medicaid—the single largest purchaser—was paying average wholesale prices. An implication of rising prices was rationing of drugs in Medicaid and subsequent access problems for Medicaid beneficiaries. For example, several studies found that states established caps on the number of prescriptions that Medicaid beneficiaries could fill,^10^, ^11^ resulting in fewer prescriptions being filled,^12^ greater use of emergency medical care,^13^ and increased admissions to nursing homes.^14^

After a long, contentious battle with pharmaceutical manufacturers and several legislative attempts to achieve savings for Medicaid,^9^ the MDRP was established relatively quickly during the final days of negotiations on the Omnibus Budget Reconciliation Act of 1990 (OBRA 90).^15^ The Bush administration was looking for savings to balance the budget and found them in a previously proposed rebate program for Medicaid. The savings would be achieved by entitling Medicaid to the “best prices” that had been presumably negotiated by other large private purchasers in the market.

However, shortly after the MDRP took effect, it became clear that the best price mechanism was having an unintended effect. Large private purchasers were in fact not achieving the lowest prices in the market. In fact, safety‐net providers, such as government‐funded public health clinics or the VA, were frequently granted the largest price reductions by manufacturers. In the presence of MDPR's best price provision, manufacturers no longer wanted to provide these significant discounts to safety‐net purchasers, even if they represented a very small percentage of the market, because those discounts would now apply to all Medicaid drug purchases. Manufacturers with price‐setting power began cancelling discounts as contracts expired.

Although policymakers had largely not anticipated this impact, some with experience in negotiating prices with manufacturers expected that prices would rise after the MDRP took effect. For example, an interviewee who had experience in negotiating prices with pharmaceutical manufacturers recalled thinking after the MDRP was authorized that “they're gonna shoot the prices up, and all of us are gonna be paying more.” A congressional staffer also recalled that the VA was upset because it felt that the MDRP's best price mechanism relied on the VA's hard‐won negotiated prices. The VA anticipated that manufacturers would increase their prices to “cost shift,” or minimize losses from the MDRP. An interviewee recalled that one manufacturer told him that “we won't be able to sell [the epilepsy drug] Dilantin to [the] VA for cheap, because now you've got Medicaid, which is a much, much bigger program.”

Still, other staffers suspected that the manufacturers’ response to the MDRP was motivated not only by economics (i.e., the cost shift) but also by a desire to undermine the rebate law. As one staffer put it, “they raised [prices] on purpose to make [a] point. It wasn't the market mechanism. It was a decision made to try to tank [the rebate law] after it happened.” To these staffers, federally funded public health clinics and the VA provided important battlefields in the war to make a point. According to another staffer, the manufacturers’ strategy was to “use the VA … and the public health [clinics as] pushback [to say] we told you this is going to cost you,” all the while “justify[ing] increas[ed] prices.”

#### A Harmed Constituency Emerges: PSHA‐Funded Clinics

Although a variety of parties were affected by the MDRP's best price provision, safety‐net clinics supported by discretionary federal grants faced a disproportionate burden of the unintended (or intended) consequences of the law. Although there was at the time—as is true today—no single definition of a “safety‐net provider,” the feature that united the safety‐net clinics harmed by the law was their reliance on discretionary federal support.^16^, ^17^, ^18^ Unlike mandatory safety‐net programs, such as Medicaid, discretionary programs are funded through the annual appropriations process and therefore have fixed budgets from year to year. Furthermore, in the presence of budget deficits, the budgets for these programs are drawn from a shrinking share of federal funds.

In the 1990s, the predominant source of support for safety‐net health care providers was appropriated grant programs authorized by the PHSA. Examples of PHSA‐funded programs included community health centers, migrant health centers, public housing clinics, family planning clinics, tuberculosis clinics, and the then‐recently created Ryan White AIDS program. Although these clinics would eventually come to rely more on Medicaid reimbursement, in the early 1990s, the Medicaid program was significantly smaller, covering roughly one‐third of the number of enrollees it does today.^19^ Therefore, federal grants were the most important source of revenue for these clinics.

An important feature of the PHSA‐funded clinics is that they are core safety‐net providers. As defined by the Institute of Medicine (now the National Academy of Medicine), core safety‐net providers are health care organizations and clinicians with a high level of demonstrated commitment to caring for uninsured and underserved patients.^18^ Specifically, core safety‐net providers are those with a legal or administrative mandate (such as a condition of federal funding) to offer services to patients regardless of their ability to pay and who care for large numbers of uninsured, underinsured, or otherwise vulnerable patient populations. Due to their payer mix, these providers had little ability to charge patients for the full amount of care. Thus, federal grant funds comprised more than half of the available funding for care in the early 1990s.^20^

In the presence of fixed federal budgets, increased drug prices from the MDRP's best price provision had the potential to jeopardize access to care. For example, the Massachusetts Family Planning Council warned that 40% to 60% increases in prices for contraceptive products between 1990 and 1991 would result in a reduction in services to their clients.^21^ A community mental health center's ten‐fold increase in prices for commonly used mental health medications over the same period was also projected to result in a reduction in both drug and nondrug services.^21^ A more specific example of the impact of rising prices between 1990 and 1991 came from the director of Maryland's Drug Treatment Program, who testified that the program's drug costs had increased by $325,000, resulting in the elimination of 125 drug treatment spots across the state.^21^ Perhaps the most striking example, the executive director of the Texas Association of Community Health Centers translated increases in drug prices between 1990 and 1991 for one specific drug, Glyburide, into approximately 4,533 fewer visits for health center patients per year.^21^ Hearing these stories from constituents worried lawmakers that scarce federal resources would be consumed by the rising price of drugs, as the Health Resources and Services Administration (HRSA) estimated that PHSA‐funded clinics spent approximately 10% of their appropriated budgets on drugs.^22^

Although it had a distinct population from the PHSA‐funded clinics and enjoyed a broad base of political support, the VA health system was in a similar predicament. Funded through appropriations and serving a group of patients—veterans—regardless of their ability to pay, hard‐fought VA discounts began to disappear after the introduction of the MDRP best price provision. Veteran's Affairs discounts stemmed from a relatively new “national contract” concept that the VA had undertaken, which shifted its procurement process from 50 contracts to just one, driving down the VA's prices. One interviewee called the VA pharmacy benefit manager program the “gold standard.” However, the impacts of OBRA 90 were jeopardizing these prices. As a result, the VA health system experienced an estimated $28 million increase in costs between 1990 and 1991, resulting in up to a 26% increase in costs at some of its VA medical centers.^23^

Given the significant political clout of the VA and the importance of the veteran population in American political discourse, addressing high drug prices became a topic of interest for several influential senators and members of congress. Senator David Pryor (D) of Arkansas, the architect of the MDRP, commissioned a report on the topic and called the pharmaceutical industry's price increases “wrong…greedy…[and] very abusive.”^24^

The PHSA‐funded clinics also had political clout, although to a lesser degree than the VA. Namely, Senator Edward “Ted” Kennedy (D‐MA), chair of the Labor, Health and Human Services, Education, and Related Agencies committee (later known as the HELP committee), was a fierce advocate for community health centers. Similarly, coming out of the HIV‐AIDS crisis, Representative Henry Waxman (D‐CA), chair of the House Energy and Commerce committee, and committee staffers had just recently authorized the Ryan White Healthcare program, which aimed to increase access to care for that patient population.^25^

### Development: The Quest to Address the Fallout From MDRP's Best Price Provision

The first proposal to fix the fallout from MDRP best price provision was initiated in 1991 by a policy staffer for Senator Kennedy. In an August 1, 1991, memo to Senator Kennedy, the staffer argued that PHSA‐funded clinics were harmed by the significant drug price increases set off by the MDRP, even though the clinics served a similar population to that served by Medicaid. Furthermore, the staffer argued that because the PHSA clinics were federally funded, “lower drug prices [would] mean that scarce dollars would stretch further.” According to an interview with the staffer, she was already aware of the unintended consequences because she had recently been a lobbyist for a consumer advocacy group and had lobbied Congress on the MDRP in 1990.

#### The First Approach: A Quick, Narrowly Focused Fix Through Appropriations

The annual appropriations process authorizes spending for the US government and directs the use of those dollars. At the time, funding for PHSA clinics was set each year through the Senate appropriations subcommittee on Labor, Health and Human Services, Education, and Related Agencies.^26^, ^27^ It therefore seemed reasonable to Senator Kennedy's staff to pursue a solution to fix the problem with MDRP's best price provision with an amendment to the committee's fiscal year 1992 appropriations bill.^28^ Senator Kennedy's staff worked with Senator Harkin (D‐IA), a member of the committee, in July 1991 to craft an amendment to the existing appropriations bill, H.R. 2707.^29^

This approach also seemed reasonable to Senator Kennedy's staff because, according to a July 23, 1991, memo from Senator Kenndy's office, another lawmaker had just used an amendment to the House appropriations subcommittee on Housing and Urban Development (HUD) and the VA to try to address the VA's drug affordability problem.^30^ The staff of Senator Barbara Mikulski (D‐MD), chair of the HUD‐VA Appropriations subcommittee, estimated that the MDRP best price provision would cost the VA $150 million in the next fiscal year. To avoid these costs, Senator Mikulski proposed an amendment to the HUD‐VA Appropriations committee's bill (H.R. 2519)^30^ in July 1991 with two changes. First, it would exempt VA prices from the MDRP best price calculation. Second, it would enable the secretary of the VA to renegotiate prices similar to those available before the MDRP.^31^ She argued that “the amendment is needed to ensure that the…VA can continue to secure the favorable prices on pharmaceutical products that it has so effectively negotiated in the past.”^32^

The amendment was included in the bill, which was passed on October 28, 1991.^33^ Senator Kennedy's staffers recalled that Senator Mikulski's approach was met with some skepticism for two reasons. First, it seemed out of scope for a VA appropriations bill, as the proposal had to do with the Medicaid program, which was not within the purview of the HUD‐VA committee. Second, it was controversial because, having just fought hard for the MDRP, lawmakers such as Senators Lloyd Bentsen (D‐TX) and James Sasser (D‐TN) of the Senate Finance committee—a committee tasked with overseeing the Medicaid program—did not want to see the cost savings from MDRP disappear.^34^ The Congressional Budget Office's approximately $700 million in estimated savings from the rebate program relied on accessing the best prices in the market, including those negotiated by the VA. The Mikulski amendment to H.R. 2519 was therefore time‐limited, giving the VA six months or until legislation could be enacted, whichever came first.^35^ This amendment was unsuccessful but provided a road map for Senator Kennedy's staff.

Learning from the reception to the Mikulski amendment, Senator Kennedy's staff articulated in internal memos from July 18, 1991, and July 24, 1991, that the draft amendment for Senator Kennedy was modeled after Senator Mikulski's amendment, but with a slight tweak. The amendment would not just ask for the PHSA‐funded clinics to be exempted from the MDRP best price provision. Instead, it would extend MDRP pricing to the clinics through a separate program. In addition, a provision was added to ensure that if the MDRP price was lower than the new program's price, the clinics would receive an additional rebate to make the discount comparable. According to another Senator Kennedy office internal memo from July 31, 1991, the Congressional Budget Office estimated that the proposal would result in savings to the clinics of $30 million in the coming fiscal year.

As with the Mikulski amendment, an internal memo from Senator Kennedy's office memo from September 13, 1991, showed that this approach was also met with some skepticism. First, Senators Bentsen and Sasser were concerned about jeopardizing the MDRP by not only exempting the clinics from the MDRP best price provision but also creating a new drug discount program just for the clinics. They signed off on the amendment but asked for a colloquy, or a written conversation, clarifying that Senator Kennedy would not make any changes to the Medicaid program, including increasing costs.

Despite the sign‐off, the amendment never made it to discussion on the full Senate floor because, according to another memo from Senator Kennedy's office from July 23, 1991, the Pharmaceutical Manufacturers Association (PMA)—the lobbying organization that would eventually become the Pharmaceutical Research and Manufacturers Association (PhRMA)—had obtained a copy. The PMA's president, Gerry Mossinghoff, strongly voiced concerns, calling the proposal an effort to “fix prices.” He also objected to the civil monetary penalties that would be imposed should the manufacturers not comply, and the technical calculation used to set drug prices for the proposed program for PHSA‐funded clinics. The PMA lobbied Senator Hatch (R‐UT), the ranking member on the Labor, Health and Human Services, Education, and Related Agencies committee and a longtime supporter of the pharmaceutical industry, to oppose the amendment.

A memo from Senator Kennedy's office from July 30, 1991, described how his staff attempted to address the PMA concerns by aligning the penalties in the new drug discount program with those in the MDRP. They also worked with PMA to resolve a technical issue on the calculation of prices in the proposed PHSA‐clinic rebate program. Although Senators Bentsen and Harkin were satisfied with this approach, and the PMA initially seemed satisfied, several manufacturers represented by the PMA objected. The PMA stated that they were opposed to the “heart” of the proposal—discounts for the PHSA clinics. Staffers from both the Senate and House recalled that manufacturers who gave significant discounts on their products, and therefore would be most impacted by the proposal, objected. Instead, the manufacturers indicated in a conversation with Senator Kennedy, described in a memo from September 13, 1991, that they would be open to solving the problem by providing voluntary discounts to the clinics, as long as Senator Kennedy abandoned his plan to propose an amendment to the Labor, Health and Human Services, Education, and Related Agencies appropriations bill.

Although a voluntary discount proposal was offered, efforts by Senator Kennedy's staff to get the manufacturers or PMA to provide details about such a proposal were unsuccessful, leading to accusations in memos from Senator Kennedy's staffers to the senator from July 30, 1991, and August 1, 1991, alleging that the PMA and the manufacturers it represented were “negotiating in bad faith.” For example, in response to PMA's claim that voluntary discounts were not possible due to anti‐trust concerns, Senator Kennedy's staff sought and received approval to offer an exemption from antitrust regulations for the manufacturers from Senator Metzenbaum (D‐OH), chair of the subcommittee on Anti‐trust, Monopolies, and Business Rights. After several rounds of proposing solutions, Senator Kennedy's staff gave up and the voluntary approach was largely abandoned. A Senator Kennedy staffer recalled that at this time, the staff recommended against the amendment to the Labor, Health and Human Services, Education, and Related Agencies appropriations bill in favor of hearings in the fall to highlight the issues of the clinics. In a September 16, 1991, letter to PMA leader Gerald Mossinghoff, Senator Kennedy warned that without a forthcoming voluntary proposal or an attempt to rework the amendment to the appropriations bill, he would be forced to pursue legislation. Legislation would also provide the opportunity for hearings recommended by Senator Kennedy's staff and could establish the rationale for legislation in the hearing record.

#### The Second Approach: The Introduction of Stand‐Alone Legislation

On September 19, 1991, Senator Kennedy introduced S. 1729,^36^ initially titled “A bill to amend the Public Health Service Act to require drug manufacturers to provide affordable prices for drugs purchased by certain entities funded under the Public Health Service Act, and for other purposes,” or by its short title, “Public Health Clinic Prudent Pharmaceutical Purchasing Act.” This bill would establish a rebate program for the PHSA clinics similar to the MDRP, including eight types of PHSA‐funded clinics, called “covered entities” and by the fact that they received “financial assistance” (Table 1). It also empowered the secretary of the Department of Health and Human Services (HHS) to negotiate prices as good as or better than those in effect before OBRA 90.

**Table 1:** Versions of 340B Proposals

Category	S. 1729 Introduced	H.R. 3405 Introduced	S. 1729 Reported	Amendment 3372 to S. 2575	H.R. 2890 Reported	H.R. 5193 Enrolled
**Sponsor and Committee**	Senator Kennedy (D‐MA), Senate Labor and Human Resources	Representative Wyden (D‐OR), House Energy and Commerce	Senator Kennedy (D‐MA), Senate Labor and Human Resources	Senator Kennedy (D‐MA) for Amendment, Cranston (D‐CA) for the bill, Senate Veterans’ Affairs Committee	Representative Montgomery (D‐MS), House Energy and Commerce	Representative Montgomery (D‐MS), House and Senate Veterans’ Affairs, House Armed Services
**Date**	Introduced 9/19/1991	Introduced 9/24/1991	Reported 3/03/1992	Offered 5/11/1992	Reported 09/22/1992	Enrolled 11/4/1992
**Pricing Mechanism**	Rebate equal to the difference between the average price paid by the covered entity and the lowest of the negotiated purchase price or the Medicaid rebate	Rebate equal to Medicaid rebate with option to negotiate lower prices	Upfront discount tied to FSS (VA price), AMP minus Medicaid rebate, or a separately negotiated purchase price, whichever is lowest	Upfront discount based on ceiling price formula: AMP minus Medicaid rebate, or a separately negotiated purchase price, whichever is lowest	Upfront discount based on ceiling price formula: AMP minus Medicaid rebate	Upfront discount based on ceiling price formula: AMP minus Medicaid rebate
**Eligible Covered Entities**						
**Community Health Centers**	Yes	Yes	Yes	Yes	Yes	Yes
**Migrant Health Centers**	Yes	Yes	Yes	Yes	Yes	Yes
**Health Centers for the Homeless**	Yes	Yes	Yes, plus 340A Housing Clinics	Yes, plus 340A Housing Clinics	Yes, plus 340A Housing Clinics	Yes, plus 340A Housing Clinics
**Alcohol/Drug and Mental Health Treatment Entities**	Yes	Yes	Yes	Yes	No	No
**Family Planning Projects**	Yes	Yes	Yes	Yes	Yes	Yes
**Ryan White CARE Act Entities**	Yes	Yes	Yes	Yes	Yes	Yes
**Black Lung Clinics**	Yes	Yes	Yes	Yes	No	Yes
**STD Clinics**	Yes	Yes	Yes	Yes	No	Yes (with certification)
**TB Clinics**	No	No	Yes	Yes	No	Yes (with certification)
**Disproportionate Share Hospitals (DSH)**	No	No	No	Yes (with minimum DSH percentage and contract with state and local government to provide indigent care)	Yes (with minimum DSH percentage and contract with state and local government to provide indigent care)	Yes (with minimum DSH percentage and contract with state and local government to provide indigent care)
**Hemophilia Treatment Centers**	No	No	No	No	Yes	Yes
**Native Hawaiian Health Centers**	No	No	No	No	No	Yes
**Tribal Clinics and Health Centers**	No	No	Yes	Yes	No	Yes
**“Resale Prohibition” AKA Diversion**	Prohibited, $25,000 civil penalty	Prohibited, $25,000 civil penalty	Prohibited, $25,000 civil penalty	Prohibited, $25,000 civil penalty	Prohibited	Prohibited
**Enforcement and Audits**	Secretary manages rebates; no specific audit provisions.	Secretary manages rebates; no specific audit provisions.	Secretary has dispute resolution authority, and both the Secretary and manufacturers have authority to conduct audits.	Secretary has dispute resolution authority, and both the Secretary and manufacturers have authority to conduct audits. Adjustments will be made if audits indicate necessity.	Secretary and manufacturers have authority to conduct audits. If noncompliant, violators are liable to the manufacturer for the value of the discount.	Secretary and manufacturers have authority to conduct audits; If noncompliant, violators are liable to the manufacturer for the value of the discount.
**Duplicate Discount Prevention**	Not mentioned	Not mentioned	Prohibits duplicate discounts; requires state notification.	Prohibited; Secretary must implement compliance mechanism.	Prohibited	Prohibited; Secretary must implement compliance mechanism.
**Prime Vendor Program**	Not mentioned	Not mentioned	Secretary, in consultation with the SVA, required to develop and implement a bid process to establish a prime vendor system.	Secretary, in consultation with the SVA, required to develop and implement a bid process to establish a prime vendor system.	Not mentioned	Secretary must establish Prime Vendor Program.
**Certification and Recertification**	Not mentioned	Not mentioned	Certification required; annual recertification	Certification required; annual recertification	Not mentioned	Certification required; annual recertification
**Use of Rebates**	Grants not reduced due to rebate receipt	Grants not reduced due to rebate receipt	Not mentioned	Not mentioned	Not mentioned	Not mentioned
**Report to Congress**	Required within 12 months of enactment: inclusion of purchased drugs, assessment of effectiveness, feasibility of FSS use, and recommendations for program improvement	Required within 12 months of enactment: inclusion of purchased drugs, assessment of effectiveness, feasibility of FSS use, and recommendations for program improvement	Required within 18 months of enactment: inclusion of drugs purchased, assessment of effectiveness, and recommendations for program	Required within 18 months of enactment: inclusion of drugs purchased, assessment of effectiveness, and recommendations for program improvement	Required within 12 months of enactment: a study evaluating the feasibility and desirability of including mental health and substance use treatment providers, STD and tuberculosis treatment clinics, and maternal and child health entities as covered entities, and analyzing drug procurement and the impacts of inclusion	Required within 12 months of enactment: a study evaluating the feasibility and desirability of including mental health and substance use treatment providers and maternal and child health entities as covered entities, and analyzing drug procurement and the impacts of inclusion

Although he did not co‐sponsor the bill, this approach was endorsed by Senator Pryor, the architect of the MDRP. And although Representative Waxman did not sponsor a bill himself, a companion bill—H.R. 3405—was introduced in the House roughly a week later by then junior lawmaker Representative Ron Wyden (D‐OR).^37^ This bill was largely the same as S. 1729 and also included the language stating that “covered entities” were defined by receiving federal “financial assistance” (Table 1).

Estimates provided by an independent academic expert and detailed in a October 11, 1991, memo to Senator Kennedy from his staff briefing him on the upcoming hearing suggested how many covered entities might be expected to participate in the program proposed by S. 1729: 216 federally qualified health centers, approximately 88 family planning programs, 133 Ryan White care clinics, 59 sexually transmitted disease clinics, 55 community mental health and drug treatment centers, and 19 public housing clinics. In total, roughly 570 centers would be expected to participate, including the 2,000 to 5,000 distinct clinical sites operated by the clinics.

Senator Kennedy's staffers recalled in interviews that they began to mobilize quickly to keep pressure on the PMA, planning a hearing that would feature public health clinics that had seen significant increases in drug prices after OBRA 90. Senator Kennedy also issued a “Dear Colleague” letter dated September 30, 1991, urging other lawmakers to sign on to the law. Specifically, the letter borrowed the phrasing from the original August 1, 1991, memo of Senator Kennedy's staffer who sought to establish a drug discount program for PHSA‐funded clinics, stating, “do not let our scarce federal dollars for healthcare be consumed by drug industry profits.” A hearing on S. 1729 was scheduled to be held on October 16, 1991.

#### The First Bipartisan Compromise: Hatch and the Splitting of the Pharmaceutical Lobby

Shortly before the October 1991 hearing on S. 1729, one manufacturer, Pfizer, broke away from the group of manufacturers who were opposing discounts to PHSA‐funded clinics to support the proposal. In a letter from October 15, 1991, the company's vice president of federal government relations, Kenneth Bowler, stated that “Pfizer Inc. supports legislation providing certain clinics assisted under the Public Health Service Act with prescription drug rebates similar to those provided to state Medicaid programs.” After stating that Pfizer could not accept price “rollbacks”, and that it wanted some technical changes to the rebate, he indicated that the manufacturer hoped that “Pfizer will be able to support this important legislation.”

Shortly after this hearing, which detailed the patient‐level impact of increased drug prices for PHSA‐funded clinics, another pharmaceutical company, Searle, reached out to discuss a voluntary discount proposal. Described in a memo to Senator Kennedy from November 5, 1991, staffers said the manufacturer expressed the desire to be proactive and avoid far‐reaching legislation such as that being proposed at the time by Senator Pryor. Senator Pryor's bill, S. 2000, would have tied pharmaceutical tax incentives to the growth of pharmaceutical prices. By January, five additional companies—Merck, Glaxo, Sytex, Astra, and Bristol Myers Squibb—would propose voluntary programs, indicating their willingness to find a solution despite the PMA's overall disapproval. Several interviewed congressional staffers recalled that one reason for the split between the PMA and these manufacturers was that these six companies were less affected by best‐price‐type proposals because they did not offer deep discounts on their drugs.

Although several pharmaceutical companies were supportive of the narrowly focused proposal to extend special pricing to PHSA‐funded clinics, the PMA and most manufacturers opposed it. In addition to the concerns raised about the appropriations‐based approach, the PMA now voiced a new concern: The existence of two rebate programs—one for Medicaid and one for the PHSA‐funded clinics—would create a “death spiral,” in which one program's best price provision would trigger the other program's best price provision until prices were driven down to zero.

Diverging opinions between the PMA and the seven manufacturers supportive of some kind of discount for PHSA clinics offered an opportunity to split the pharmaceutical manufacturers. Senator Kennedy's staffers recalled at this point that they would also need a co‐sponsor with close ties to the pharmaceutical industry to ultimately get the bill passed. In November, Senator Kennedy reached out to Senator Hatch to seek his support for the bill. Senator Kennedy's staffers stated in an internal memo from November 13, 1991, that Senator Hatch was surprisingly receptive. Senator Hatch was a longtime supporter of the pharmaceutical industry, helping to co‐author legislation that affected generic approval, patent length, and the establishment of orphan drugs.^38^ Because of these relationships, Senator Kennedy's staffers felt he could persuade manufacturers to agree to passing S. 1729. However, staffers recalled expressing skepticism because Senator Hatch had not been supportive of the amendment to the appropriations bill.

Surprisingly, by the end of 1991, something had changed and Senator Hatch became supportive of the bill. Although the exact reason is unknown, both Senator Kennedy and Senator Hatch's staffers thought it was likely that Senator Hatch was swayed by a personal connection who was supportive of the law: Kenneth Bowler, the president of federal government relations at Pfizer. Senator Hatch and Bowler were friends, and Bowler's father had worked on Senator Hatch's first campaign for a seat in the Senate.^39^ After Bowler, who favored the bill, made a personal appeal, Senator Hatch became supportive of the law and interested in negotiating. His initial proposal, according to a memo to Senator Kennedy from November 11, 1991, was to grant the PHSA‐funded clinics a flat percentage rebate instead of extending best price rebates, similar to the MDRP. However, this approach was overruled by Senator Kennedy's office because it would have interacted negatively with the MDRP.

Another proposal that was contemplated in discussions between Senator Hatch and the Senator Kennedy staffers was to let the PHSA clinics “ride” on the VA prices. In other words, PHSA‐funded clinics could access the same prices negotiated by the VA. Although these prices were seen as not as competitive as MDRP prices, they represented a significant discount. A memo from Senator Kennedy's office from November 20, 1991, stated that the approach quickly gained the support of eight pharmaceutical manufacturers. However, a November 22, 1991, memo from Senator Kennedy's office stated that Senator Hatch sought three conditions for his ultimate approval of the proposal: a 20% cap on the VA‐based discounts, the “deletion” of the VA prices after two years, and the elimination of the 10% discount for generic drugs.

The addition of these criteria was met with skepticism by Senator Kennedy's staffers, according to the same Kennedy office internal memo from November 22, 1991. Although it was a bipartisan effort, Senator Kennedy's staff tried to negotiate with Senator Hatch on the cap. When that effort was unsuccessful, Senator Kennedy's staff worried that Senator Hatch was stalling because a few drug companies would not compromise, according to internal memos from the end of November 1991.

But while Senator Kennedy's staff believed they had reached an impasse, Senator Hatch had begun to do what can only be described as “reverse lobbying” of the drug manufacturers. According to a former staffer, Senator Hatch leveraged the lobbying firm of a former chief of staff,^40^ instructing them to relay a message to their clients: Senator Hatch wants you to support this bill. According to a lobbyist and several staffers, this message was likely compelling to the manufacturers given Senator Hatch's prominent role on the committees of importance to the drug industry—Labor and Human Resources, which oversaw the Food and Drug Administration, and Finance, which presided over tax policy.

By December, Senator Hatch's efforts brought a critical mass of pharmaceutical manufacturers to the table. At a meeting of 33 chief executive officers from the PMA member companies, 30 endorsed a proposal to extend discounts to PHSA clinics. In January, Senator Kennedy's and Senator Hatch's staff began to mark up the bill based on a compromise negotiated by Senator Hatch, according to a memo from Senator Kennedy's office from January 24, 1992. This compromise proposal consisted of extending VA prices to the PHSA clinics with a minimum 12.5% discount not to exceed 25%. The cap would be set at a dollar amount that would increase with inflation rather than stay at a fixed rebate percentage.

The compromise bill was also rewritten to include statutory audit authority for both the HHS secretary and manufacturers to prevent diversion of the drugs to non‐PHSA‐funded clinics.^41^ It also included a “duplicate discount” provision,^41^ which would guarantee that manufacturers would not be required to pay both a discount through the new program and the MDRP on the same drug. At this point, Senator Kennedy staffers recalled they expected no opposition from the pharmaceutical manufacturers, and the bill was ordered to be reported from the committee “favorably,” which indicated a majority of support for the proposal.^42^

The markup also added several new federally supported clinics to the list of covered entities. In addition to the original list of covered entities from S. 1729 and H.R. 3405, the PMA compromise bill included tuberculosis programs receiving assistance under Section 317E of the PHSA and an entity not funded directly by the PHSA: “a non‐Federal entity authorized under the Indian Self‐Determination Act.” In other words, clinics operated by Tribal authorities of the Indian Health Service.^41^ (see Table 1.) However, the Tribal clinics could easily be linked to the PHSA‐funded clinics under the umbrella of receiving federal financial assistance.

Despite the initial confidence expressed by the Senator Kennedy staffers over the emerging compromise, it was soon apparent that the threat of manufacturers withdrawing from the deal was still significant. One congressional staffer recalled that there was a particular fear that one manufacturer located in North Carolina would lobby Senator Jesse Helms (R‐NC) to oppose the bill. One solution staffers hoped might save the bill was linking the solution for the public health clinics to the solution for the VA, a constituency with significant political clout.

#### The Third Approach: Linking the PHSA Clinics and the VA

A coalition between senators on the Senate Labor, Health and Human Services, Education, and Related Agencies committee and the Senate VA committee—calling themselves the “Gang of Five”—began to form in February of 1992, as articulated in a memo from Senator Kennedy's office. The gang—composed of Senators Alan Cranston (D‐CA), Kennedy, Mikulski, Pryor, and Jay Rockefeller (D‐WV), worked on a proposal in the spring and summer of 1992 to guarantee minimum discounts to both the VA and the PHSA‐funded clinics. On October 1, 1992, they introduced their proposal as an amendment to S. 2575,^43^ an existing bill on health care issues related to the VA. Specifically, Senator Cranston amended the bill to establish a mechanism to secure discounts for the VA and the Department of Defense and enforce it through a “master agreement.” The agreement would require manufacturers to honor price concessions with these federal purchasers if they wanted drugs to be reimbursed by federal government purchasers, including Medicaid. Shortly after, Senator Kennedy amended Senator Cranston's amendment with Amendment No. 3372, co‐sponsored by Senator Hatch, to include the PHSA clinics in the group of federal purchasers^44^ (see Table 1).

This language from the Cranston–Kennedy amendment to S. 2575 was ultimately substituted into the House's VA bill on health care (H.R. 5193)^45^ when it was debated by the Senate in October 1992^45^ (see Table 1). At this point, a stable triangle was proposed: In order to have drugs reimbursed by the Medicaid program, manufacturers would be required to offer MDRP‐comparable discounts to the VA and PHSA‐funded clinics. Furthermore, the prices for both the VA and the PHSA‐funded clinics would be excluded from the MDRP's best price provision, preventing further price increases.

In addition to linking the PHSA‐clinic discount program to the VA and Medicaid, changes were introduced to the program in the process of reporting S. 2575. In the Senate and House proposals, PHSA‐clinic discounts had mirrored the MDRP's post‐transaction rebate structure. In other words, discounts would be applied after drugs had been dispensed or administered to patients. However, in S. 2575, the discount was rewritten as an up‐front, or pre‐transaction, discount available at the time of purchase. According to interviews with congressional staffers and advocates, this change was made because Senator Kennedy's staffers had spoken to advocates for PHSA‐funded clinics and learned that a rebate program would pose significant challenges that the clinics would be unable to meet given their overall lack of administrative resources and federally appropriated budgets.

At this point, the proposed mechanism linking HHS agreements over the MDRP, VA master agreements, and PHSA‐specific purchasing agreements developed by the Gang of Five began to move into an existing legislative proposal in the House. Representative Sonny Montgomery (D‐MS), chair of the House Veterans Affairs committee, introduced H.R. 2890 in July of the previous year to make permanent the protections from the MDRP's best price provision for the VA won by Senator Mikulski.^46^ (See Table 1.) The bill, called the Medicaid and Department of Veterans Affairs Drug Rebate Amendments, sought to exempt the VA from MDRP's best price calculations, empower the secretary to renegotiate prices to pre‐1990 levels, and require manufacturers to honor discounts to the VA as a condition for having their drugs covered by Medicaid.

According to interviews with house staffers, explicitly linking VA discounts to the MDRP approach was not popular with either Representative Waxman (chair of the Energy and Commerce committee) or Senator Bentsen (chair of the Finance committee) in 1991. Both legislators were hesitant to jeopardize access to drugs for Medicaid patients if manufacturers did not comply. However, Representative Montgomery, as the chair of the politically powerful VA committee, insisted after Senator Bentsen's and Representative Waxman's staff balked at the proposal. Shortly after, the staffer recalled, Senator Bentsen's and Representative Waxman's offices were inundated with complaints from veterans groups, who quickly changed the lawmakers’ minds.

Despite the support of veterans, the successful linkage of VA discounts and the MDRP, and a markup of H.R. 2890 in the Veterans committee in November 1991, Montgomery's bill was stalled for many months. However, Representative Waxman began to express interest in the impact of the MDRP on the PHSA‐funded clinics in the summer of 1992. As a house staffer recalled, “he had an interest in the public health side of the health subcommittee's jurisdiction and wanted to make sure they were able to do what they were funded to do.” Giving the clinics the same net prices as Medicaid would protect the clinics from drug price increases. Eventually, Montgomery's bill was referred to the House Energy and Commerce committee, where Waxman held hearings on both H.R. 2890 and H.R. 3405 in July of 1992.

At this point, language explicitly linking the MDRP and the VA from H.R. 2890 was replaced with the Gang of Five's compromise language from S. 2575. In its report from September 1992, the House Energy and Commerce committee stated:

The Committee is persuaded that, without intervention, the [VA] and federally funded clinics may continue to experience substantial drug price increases as manufacturers try to limit their rebates to Medicaid. In the view of the Committee, the Federal government simply cannot continue to allow the [VA], federally funded clinics and their patients to remain unprotected against manufacturer price increases.^47^

Although the report framed the policy proposal as addressing affordability problems for federal grantees, a staffer recalled that “as other people heard and understood what we were trying to do, and they saw the value of [discounts] for their covered entity population, whether it was the hospitals or whoever, they also came along and wanted to be included…so there [were] a lot of politics.” One of these groups advocating for inclusion in the PHSA‐funded clinic discount program was public hospitals.^48^

### Compromise: The Inclusion of Hospitals in 340B

#### Public Hospitals: Not Dependent on Federal Support but Similar Mission to PHSA Clinics

As the proposals in H.R. 5193 and H.R. 2890 were coming together, the list of original covered entities that were included in H.R. 3405 and S. 1729 was growing beyond the narrow scope of PHSA‐funded clinics. Among the early motivations for introducing a remedy for the MDRP best price provision for the PHSA‐funded clinics was policymakers’ desire to use scarce, appropriated federal funds wisely. Unlike Medicaid, which is an entitlement, PHSA funding was capped, determined annually by appropriation committees in Congress, and therefore vulnerable to budget cuts. Hence the introduction of the proposed discount program to make PHSA‐funded clinics’ “scarce” federally appropriated dollars “stretch further,” as the Kennedy staffer wrote in her original memo from August 1, 1991.

In Representative Waxman's hearing on July 31, 1992, representatives from public hospitals, including MacGregor Day of Parkland Memorial Hospital speaking on behalf of the National Association of Public Hospitals (NAPH), testified that member hospitals were excluded from remediations from the fallout from MDRP's best price provision despite serving large numbers of low‐income and uninsured patients. In his testimony, he urged Congress to extend drug discounts to these institutions, arguing that without access to rebates, public hospitals were paying inflated prices for essential outpatient drugs while for‐profit purchasers received steep discounts—an inequity that strained hospital budgets and compromised care for vulnerable populations.^49^

The problems faced by NAPH‐member hospitals posed an important question: Should the new discount programs be constrained by focusing on using “scarce” *federal* dollars wisely, or could the logic be expanded to other core safety‐net providers that did similar work and faced similarly fixed, yet state and locally funded, budgets? Many of these hospitals faced requirements to provide free and discounted care, but—like the PHSA‐funded clinics and the VA—their fixed budgets were being squeezed by increasing drug prices set off by the MDRP. As one Kennedy staffer recalled, policymakers were interested in helping public hospitals because even though they “got a lot of Medicaid money, they also [did] a lot of uncompensated care and had to rely on state and local and charitable fundraising.… They had a finite amount of money and they're not being reimbursed, so we should allow them to conserve their resources to be used for other purposes.” Still another staffer recalled that public hospitals were supported by taxpayers, so extending the discounts to public hospitals could ultimately save taxpayers money.

Indeed, the public hospitals—represented by NAPH—had already been advocating for relief from MDRP's best price provision. They had joined a “best price coalition” that included other private buyers such as health maintenance organizations and group purchasing organizations, according to an interviewee. However, when NAPH realized it could successfully lobby for inclusion in the new PHSA‐clinic focused discount program, it split from this coalition, which was ultimately unsuccessful in persuading lawmakers to intervene on its behalf. The inclusion of public hospitals was, as one interviewed staffer recalled, the first departure from 340B's more narrow focus on PHSA‐funded clinics.

The possibility of NAPH member hospitals to participate in 340B was facilitated by the receptiveness of Representative Waxman, who represented a district that included Los Angeles, California, and therefore one of the largest public hospitals in the country—Los Angeles County Hospital. Although these hospitals did not receive significant funding from the PHSA, staffers of the committee acknowledged that these hospitals were under similar constraints as the PHSA clinics. However, one staffer recalled that Representative Waxman “was willing to put in a couple of hospitals” but there was some skepticism because the public hospitals had just gotten an exemption from provider taxes and were benefiting significantly from Medicaid disproportionate share hospital (DSH) payments at the time. In addition, another staffer recalled that Kennedy was receptive to the proposal because there were connections between Senator Kennedy's office and NAPH: One of his former staffers was now it's head.

Although the argument was made to include public hospitals in the program despite their lack of reliance on appropriated federal funding, some federally funded clinics were excluded because their budgets were set through appropriations. For example, the Senate committee reported bill included community mental health centers and drug and alcohol treatment centers, which were also struggling with rising drug prices as discussed at the hearing on S. 1279, the original legislative proposal to provide relief from high drug prices to PHSA‐funded clinics. However, the Alcohol, Drug Abuse, and Mental Health Services Reorganization Act of 1992 turned both types of clinics’ funding into state block grants. Ultimately, these clinics were kept out of the list of covered entities because a minority staffer to the Energy and Commerce committee argued that the clinics no longer had a direct link to federal oversight, as reported during a key informant interview. Although they were excluded, the Kennedy staffer who had originally identified the problem for PHSA‐funded clinics sought to eventually add them to the list of covered entities by requiring a study on the feasibility of their inclusion in the bill that would ultimately become the authorizing legislation for 340B.

Although the link between fixed federal funding and fixed state and local funding was plausible, the inclusion of nonprofit hospitals to accommodate this trend unintentionally opened a crucial loophole: In the absence of robust criteria for the definition of core safety‐net status for hospitals (such as public ownership), it would be very difficult to separate the public hospitals that had recently converted to nonprofits from nonprofits with little to no safety‐net mission.

#### The Search for Objective Hospital Eligibility Criteria for Public Hospitals

Once a decision had been made to include public hospitals in the list of covered entities, representatives of NAPH worked closely with House Energy and Commerce staff to identify the approximately 200 member hospitals, which included both public hospitals and private nonprofit hospitals that had recently converted from public status.^50^ According to a key informant interview, it was important to include formerly public, nonprofit hospitals because such conversions had become a popular way to make public hospitals more nimble and competitive with private hospitals in the presence of significant changes to Medicare in the 1980s. Specifically, public hospitals were thought to gain flexibility and easier access to capital by abandoning traditional public governance, and attracting new patient populations. However, many of these hospitals sought to maintain their original mandate to provide access to care regardless of ability to pay.^50^ They were for all intents and purposes still public hospitals.

To respond to these new trends, H.R. 2890 included in its list of covered entities a hospital that the HHS secretary certifies is

owned or operated by a unit of State or local government, is a public or private nonprofit corporation which is formally granted governmental powers by a unit of State or local government, or is a private nonprofit hospital which has a contract with State or local government to provide health care services to low income individuals who are not entitled to benefits under Medicare or Medicaid.^48^

This language was broad to accommodate the many types of arrangements pursued for public hospitals, including acquisition, lease, or management by a nonprofit hospital; public–private partnerships; or quasi‐governmental structures such as public benefit corporations.^50^

In addition to the aforementioned ownership criteria, staffers sought to use an additional criterion that would further separate the formerly public hospials from non‐profit hospitals without high burdens of uncompensated care. A house staffer recalled that they were looking for “things that were in plan already, operationalized, not subject to manipulation, [and] reasonably objective.” The same staffer speculated that the hospitals suggested the adjusted DSH patient percentage would likely identify most hospitals policymakers sought to protect—the NAPH member hospitals. Developed by Congress to assess safety‐net hospital status for the purpose of allocating Medicare DSH payments, the DSH patient percentage is defined as the sum of the share of inpatient Medicare beneficiaries eligible for supplemental security income and the share of total inpatient volume that is eligible for Medicaid. After adjustment for hospital size and geographic location, the adjusted DSH patient percentage was used as a multiplier to calculate hospitals' total DSH payment.^51^ Although the staffers believed it reflected uncompensated care provisions, the Congressional Budget Office had recently acknowledged that its relationship to uncompensated care was weak and should eventually be replaced with a better measure.^52^

As staffers and advocates involved in setting the criteria recalled, “the question [was] which hospitals were going to qualify, and I remember us running lots of spreadsheets…trying to figure out where to put the DSH adjustment.” According to Medicare hospital cost report data from 1991–1992, the average adjusted DSH patient percentage of NAPH member hospitals was 25%, ranging from a minimum of 13% to a maximum of 45%, with more than 10% of NAPH hospitals having adjusted DSH patient percentages in excess of 40%. Multiple interviewees recalled that the selected criterion was supposed to qualify about 200 hospitals, corresponding to NAPH's membership at the time. As one stated, “the emphasis was [on] trying to keep the number of hospitals relatively small.” No interviewees could recall the original adjusted DSH percentage eligibility level set to qualify these 200 hospitals. However, data from 1991–1992 suggest the original eligibility level could have been 13% based on the minimum adjusted DSH patient percentages of probable NAPH member hospitals in that year.^53^

#### Tweaking the DSH Eligibility Cutoff to Gain Bipartisan Support

As staffers grappled with how to write eligibility criteria to qualify the roughly 200 NAPH member hospitals, including both public and recently converted public hospitals, bipartisan compromises were made to ultimately secure passage of the legislation. According to interviews with several staffers, the first compromise was made with Representative Bliley from Virginia. He agreed to support the proposal as long as the University of Virginia's nonprofit hospital was allowed to participate. To accommodate this request, one staffer recalled that they had to “tweak” the eligibility threshold, lowering it so it would include the University of Virginia's hospital. Using data provided by the HHS, staffers identified the minimum cutoff necessary to include the University of Virginia (12.5%) and included it in H.R. 2890 when the bill was reported in the House in September 1992 (see Table 1.)

Although House Energy and Commerce staffers were fine with introducing hospitals to the program, Senate staffers on the Labor, Health and Human Services, Education, and Related Agencies committee were not, according to interviews with both Senate and House staffers. They worried that opening the door to hospitals could make the bipartisan dealmaking in the Senate fall apart. Thinking the inclusion of the hospitals would be a deal killer for Senator Hatch, who was closely aligned with the pharmaceutical industry, they were surprised to learn that Hatch was happy to include hospitals, provided that the University of Utah's hospital could also qualify. To accommodate Senator Hatch, staffers went back to the list provided by HHS and found the new necessary minimum eligibility criterion: 11.75%.

At this point, H.R. 2890, the bill that had come through Representative Waxman's House Energy and Commerce subcommittee, was abandoned, but its language on hospitals as covered entities had made it, along with the proposal linking the VA, the MDRP, and the PHSA‐funded clinic into the House VA bill, H.R. 5193, the Veterans Health Care Act of 1992. Now the 12.5% threshold in H.R. 5193 was lowered to 11.75% to accommodate Senator Hatch. However, this change was made late in the process, appearing only in the enrolled version of the bill.^45^

Although staffers interviewed did not realize it at the time, substantially more hospitals than initially targeted for inclusion would meet the adjusted DSH patient percentage threshold after the two bipartisan tweaks. According to data on the adjusted DSH patient percentage from 1991–1992, 352 public and nonprofit hospitals had an adjusted DSH patient percentage that exceeded the lowest NAPH member's adjusted DSH patient percentage (13%), including 152 public hospitals and 200 nonprofits. Lowering the criterion to 11.75% added another 27 public and 64 nonprofit hospitals, for a total of 91 additional hospitals.^53^

### Passage and Early Implementation: Senator Kennedy's Initial Proposal Becomes a Reality

The Veteran's Health Care Act of 1992^47^ would eventually be passed on the last day of the 102nd Congress and signed into law on November 4, 1992, by George H.W. Bush. This law, which created a statutory discount program for PHSA clinics and other federal grantee safety‐net providers and exempted these providers from MDRP's best price calculations, would come to be known as “340B” by Waxman's staff—named for the section of the PHSA it would create and tied closely to the community health center sections of the PHSA.

#### Early Challenges for the HRSA

Although the VA moved fairly quickly to implement the VA‐specific aspects of H.R. 5193, the agency tasked with starting the program, HRSA, was slow to begin implementation of the 340B program. A new Office of Pharmaceutical Affairs in HRSA had to be created and staffed, and eligible covered entities would need to be identified and enrolled.^54^ Although it was estimated that roughly 600 grantees, operating approximately 2,000 clinics, would be eligible under S. 1729, it was unclear how many hospitals would be eligible to participate. As hospitals were added in later interations of the proposal, the new Office struggled with how to identify them and relied on the spreadsheet congressional staffers in the House had used to select the 11.75% DSH patient percentage eligibility criterion. An interviewee recalled that “everybody involved with 340B at HRSA kept saying, we've never worked with hospitals…they've always been regulated by CMS.” Indeed, interviewees recalled that HRSA was originally tasked with regulating 340B because the program was originally designed for grantees of the PHSA.

As HRSA struggled to identify and contact potentially eligible hospitals, the agency began to rely on groups representing hospitals. According to one interviewee, one of these groups began to host meetings to encourage hospitals to participate in the program. This group would eventually become 340BHealth, the membership organization that advocates for 340B hospitals and health systems.^55^

#### Clinton Health Reform, Midterms, and the Interruption of the Iterative Policy Process

As the implementation of 340B was occurring, the health reform movement in the administration of President Bill Clinton was becoming an important new push for lawmakers, as achieving universal coverage had long been a goal of powerful Democrats. Although the scope of the nascent 340B program was beginning to diverge from Congress's original intentions, staffers from both parties noted that Clinton health reform occupied them all through 1993 and 1994 and that the Clinton Health Task Force, on which many democratic staffers served, was a “completely absorbing process.” Acknowledging that implementation was a challenge to HRSA, one staffer recalled that he hoped that as the administration changed from Bush to Clinton, “that maybe the implementation of both the [Medicaid] drug rebate program and the 340B program might be better.” However, as congressional leadership—as well as priorities—changed in 1994, health programs were no longer a priority and lawmakers did not return to 340B. Thus, there was no chance to assess how well the program aligned with Congress's original intentions or to adjust it to better align it with those intentions.

Perhaps the interaction of HRSA's new role, Clinton's health reform, and changing congressional priorities were all factors in the lack of refinement and adjustment of the program through an iterative process—identifying unintended consequences of policy and then proposing and adopting solutions through the legislative process—which had become precedent for health policymakers. One staffer stated that the legislative style of his committee assumed “continuing legislative ability to refine and adjust” and after anticipating vulnerabilities, planning to “come back in a couple of years…and fix it.” Indeed, the Veterans Health Care Act of 1992 was such a return. The VA and 340B programs would not have existed unless the MDRP's best price provision had eliminated discounts for federal payers on appropriated budgets. Thus, the lack of the critical iterative process is another illuminative section of this program's history that helps to explain where it is today.

## Discussion

In our analysis of 19 structured interviews with 18 key informants and a review of more than 175 primary source documents, we conclude that Congress had two intentions for 340B when it was created in 1992. First, lawmakers wanted to quickly address the unintended consequence of the MDRP's best price provision, which inadvertently raised costs for core safety‐net providers. These providers—federally funded safety‐net clinics and the VA—previously purchased small quantities of drugs at steep discounts. To prevent these prices from becoming “best prices” and possibly to undermine the rebate law, manufacturers began to cancel their steep discounts. By removing both VA purchased drugs and drugs purchased by federal grantees under 340B, Congress shut down this unintended program interaction.

Second, lawmakers sought to preserve the budgets of core safety‐net providers largely funded through discretionary federal spending that were required to offer free or reduced‐price drugs regardless of patients’ ability to pay. Even with an exemption from Medicaid's “best price,” the rising costs of drugs were straining their resources, forcing cutbacks in discounted medications and other care. By extending Medicaid rebate‐like discounts to core safety‐net providers, Congress entitled a group responsible for supporting safety‐net care with its own special drug purchasing program (similar to Medicaid and the VA) so that they could continue to provide care to patients regardless of cost and reinvest savings to serve more patients and more low‐income communities.

Public hospitals, though not federally funded, were included because they faced similar affordability challenges. However, in the process of making bipartisan deals to support the policy, lawmakers ended up introducing indiscriminate eligibility criteria that qualified specific hospitals of interest to key Republicans but also made many other nonprofit hospitals—now arguably 340B's largest beneficiaries—part of the program. Although they had large inpatient Medicaid shares, which qualified them for the program, they were not core safety‐net providers and did not have a requirement to share discounts with patients who could not pay. In addition, the presence of patients with traditional insurance created an opportunity not just to lower the loss associated with providing free and discounted drug care but also to generate a profit for the entity, through collecting reimbursement higher than the 340B price. As one interviewee put it, the difference between reimbursement and discounted price “became a method of finance, but it was never intended [to be].”

In recent debates about Congress's original intentions for the program, the words “stretch scarce federal resources” have often been used to justify the program as a method of finance. However, these debates missed a critical nuance. The words “stretch scarce federal resources” were meant to motivate action to give relief from escalating drug prices for a particular type of provider: those heavily dependent on appropriated federal, state, and local grants, and with a mission to provide free and discounted drugs to patients. With relatively few insured patients drawn from predominantly low‐income populations, lawmakers wanted to give flexibility to these providers with the goal of increasing access to drugs for patients. It did not occur to them that these words would be used to justify the opposite for nonprofit hospitals—charging patients prices significantly above the discounted 340B price and fighting to prevent any requirements to share discounts with patients.

In lockstep with the unanticipated use of the program as a method of finance, the need to define a target indigent patient population did not occur to the staffers or lawmakers working on the proposals because both the VA and the PHSA clinics largely had well‐defined patient populations and a requirement to provide care to these populations free of charge or at a significant discount. However, the introduction of DSH‐qualifying nonprofit hospitals complicated things. These hospitals did not specialize in serving a population that was, by definition, unable to access care—because of either income or stigma—and they had payer mixes that included patients covered by commercial insurance. Similarly, the hemophilia clinics had many insured patients. However, these issues were not addressed at the time the Energy and Commerce committee that added these entities to the list of eligible participants.

Although Congress's intention may have changed since the passing of the 340B program, the focus of the analysis was to better understand the intent of those who curated the 340B statutory language. In addition to clarifying Congress's original intentions, the analysis provides several other lessons for health policy. First, although the program was designed to help core safety‐net providers, it, like other programs made to support the safety‐net, now primarily benefits non‐safety‐net providers. The introduction of noncore safety‐net providers can be attributed to the inclusion of the DSH‐based criterion and a minimum level set to qualify specific nonprofit hospitals in the constituencies of ranking members on key committees: legislators Representative Bliley and Senator Hatch. Using data from 1991 and 1992, we calculated that the cutoff of 11.75% also would have qualified more than 500 nonprofit general acute care hospitals.^53^

Other programs, such as Medicare and Medicaid DSH payments, have become less well‐targeted over time.^56^, ^57^ Poor targeting has much to do with the lack of a uniform definition of safety‐net providers; many of the commonly used definitions identify different providers and also disproportionately qualify hospitals over other, nonhospital providers that provide significant amounts of safety‐net care. For this reason, the Institute of Medicine's claim that the US safety net was “intact but endangered” is still relevant decades later.^18^

Second, policymaking is an iterative process and requires policymakers and their staff to experience the program as it is implemented, hear from affected constituencies, and take action to revise policy. Indeed, if the staffers and policymakers who had been working on Medicaid drug rebates, VA discounts, and 340B discounts from 1990–1992 became aware of the problems with 340B's targeting and lack of benefits for patients, they may have sought to fix them. In addition, if policymakers had returned to 340B's statute, they would have been able to put bounds around aspects of the program, such as contract pharmacies, that are today vulnerable because they are purely based on HRSA's interpretation of a vague statute. The original statute simply states that drugs shall not be “resold” to patients who are not patients of the covered entity. Contract pharmacies were created by HRSA several years after the program started in response to the fact that many grantees that were eligible for the program were unable to access the discounts because they did not have an in‐house pharmacy. To increase access to 340B‐discounted drugs, HRSA decided to allow covered entities to establish one contract with a pharmacy in the community.^58^

Yet shortly, the covered entities and HRSA would discover that contract pharmacy arrangements conflicted with typical retail pharmacy distribution models for large chains. Specifically, an interviewee noted that chains told them they made choices about how to distribute drugs at a regional level, not an individual pharmacy level. Therefore, contracts between covered entities and individual pharmacies to purchase and ship 340B‐discounted drugs to individual pharmacies were administratively inconvenient for the contracting pharmacy chains. Wanting to increase safety‐net provider participation in 340B, HRSA created the “alternative methods demonstration project, which allowed covered entities to have more than one contract pharmacy.”^59^ This demonstration project would eventually lead to HRSA's guidance in 2010 that allowed covered entities to have an *unlimited* number of contracts with retail pharmacies.^60^ This is a critical example of why the iterative process is so important. HRSA was acting off of a vague statute, which led to a growth in contract pharmacies that could never have been anticipated by those involved in the original construction of the program.

Third, although manufacturers are generally opponents of 340B, the program would not have existed if manufacturers had agreed to a voluntary discount. Senator Kennedy's staffers made attempts to find a solution that did not require a statutory discount program. However, the trade association for the manufacturers—PMA—did not want to compromise. Interestingly, a number of manufacturers were supportive of both voluntary policies and even 340B in its original form that did not include hospitals.

Fourth, the policymaking process during the creation of 340B can be characterized by the “iron triangle,” the set of interdependent relationships between staff of congressional committees (e.g., Energy and Commerce, VA), representatives of special interest groups (e.g., PMA, NAPH), and bureaucrats who work as regulatory agencies (e.g., HRSA).^61^ These relationships benefit interest groups by giving them close proximity to both lawmakers and the agencies that implement the programs, they benefit congressional staffers by facilitating the passage of desired policies, and they benefit the bureaucracy by securing appropriations and public support.^62^

Fifth, although HRSA's guidance hewed relatively close to lawmakers’ intent in 1992, subsequent policy choices began to deviate from the original goals as the program grew. Several factors explain this deviation. First, Congress left many program features undefined, creating ambiguity that could create significant potential pressure for HRSA to side with the interpretation of interest groups. Second, HRSA's lack of resources necessitated that it rely on industry groups. The enabling legislation included no money for enforcement, and HRSA recently stated that it lacks the authority to enforce its own guidance.^63^

Finally, as health policy is increasingly set in the courts, it is important to understand Congress's original intentions for 340B. Given the brevity of 340B's statute and the many issues it remains silent on—the patient definition, contract pharmacy, interactions between 340B and other drug discount programs in the US health care system, whether 340B should be treated as a source of revenue, and how those revenues should be used—judges may rely on the historical context in which the statute arose to interpret its meaning. This qualitative analysis of the legislative history—by illustrating the importance of the MDRP, the abandoned approaches, and the bipartisan compromises that shaped it along the way—can clarify the problem that 340B was meant to solve: supporting core safety‐net providers and making drugs affordable for their patients.

## Funding/Support

The Commonwealth Fund

## Conflict of Interest Statement

The authors report no conflicts of interest. Nikpay received unrelated funding from the National Institutes of Health, Arnold Ventures, the Centers for Medicare and Medicaid Services, the Minnesota Department of Health, and unrelated consulting payments from Analysis Group and Health Affairs Scholar during the time this study was conducted.

## Supporting information



Table A.1 Interview Candidates and Interview Status
Table A.2 Inventory of Primary Source Documents Referenced in the Article
