# Supplementary material for: Stretching Scarce Authorizing Legislation as Far as Possible: A Legislative History of the 340B Drug Pricing Program
Source: Milbank Q. 2026 May 20:10.1111/1468-0009.70090. Online ahead of print. doi: 10.1111/1468-0009.70090 (PMC13560479; doi:10.1111/1468-0009.70090)
Supplement: Supplementary file 1 [file MILQ-9999-0-s001.docx]

**Appendix**

**Table A.1 Interview Candidates and Interview Status**

| Name | Position 1991-1992 |
| --- | --- |
| Andy Schneider, JD | Energy and Commerce Committee, Lead Majority Staffer on 340B Enactment |
| Bill Corr, JD | Staff Director, Anit-Trust Subcommittee, Judiciary Committee |
| Bill Von Oehsen, JD | Counsel for the National Association of Public Hospitals |
| Chris Jennings, JD | Senate Aging Committee Staffer |
| David Schulke | Health Legislative Assistant for Representative Wyden, Former staffer for Senator Pryor |
| Howard Cohen, PhD, JD | House Staffer: Energy and Commerce Committee, lead minority staffer |
| Jim Cooper, JD | Representative, Democrat, Tennessee |
| John Coster, PhD | Senate Aging Committee, Lead Staffer on Drug Issues (Including Medicaid Rebate) |
| John Haddow | Labor and Human Resources Committee, Lead Minority Staffer on 340B (and staffer for Senator Hatch) |
| Jose Camacho, JD | Executive Director, Texas Association of Community Health Centers and Founder of FQHC Drug Purchasing Cooperative |
| Marsha Simon, PhD | Legislative staffer for Senator Edward Kennedy, Original drafter of legislation that became 340B |
| REDACTED | Government relations for REDACTED pharmaceutical company |
| Roman Romani, PhD | Lobbyist |
| Ron Weich, JD | Labor and Human Resources Committee, Lead Majority Staffer for Substance Abuse and Mental Health Programs |
| Steve Schondelmeyer, PharmD, PhD | Professor and Economist, University of Minnesota College of Pharmacy |
| Thomas Scully, JD | Deputy director of the Office of Management and Budget |
| Tim Westmorland, JD | Energy and Commerce Committee, Lead Majority Staffer on the Ryan White Program |
| Tony Principi, JD | Deputy Secretary of Veterans Affairs |

The original list of interview participants included 42 candidates. 19 interviews were conducted, 2 candidates declined to be interviewed, 7 candidates never replied to our attempts to reach them, and finally 16 candidates were not interviewed because we could not identify contact information, including one who had died. One candidate was interviewed twice.

**Table A.2 Inventory of Primary Source Documents Referenced in the Article**

| Description | Date |
| --- | --- |
| Developments in Aging: 1990 Volume 1, S. Rep. No. 102-28, 102nd Cong. (1991). | 02/28/1990 |
| Memorandum from Marsha Simon on New Developments on Drug Prices to Senator Kennedy | 07/18/1991 |
| Memorandum from Marsha Simon on the Amendment to the Labor HHS Appropriations bill on drug discounts for Public Health Service Act clinics to Senator Kennedy | 07/23/1991 |
| Memorandum from Marsha Simon on the Amendment to the Labor HHS Appropriations bill on drug discounts for Public Health Service Act clinics to Senator Kennedy | 7/23/1991 |
| Memorandum from Marsha Simon of Talking Points: Senator Hatch and the Drug Price Amendment | 07/31/1991 |
| Memorandum from Marsha Simon and Mark on Drug Pricing and the Pharmaceutical Manufacturers Association (PMA) -- call Gerry Mossinghoff again before recess to Senator Kennedy | 08/01/1991 |
| Memorandum from Marsha Simon on the Public Health Service Act Clinics: The Need for Drug Price Relief | 08/01/1991 |
| Colloquy among Senator Bentsen, Senator Kennedy and Senator Sasser | 09/1991 |
| Memorandum from Marsha Simon and Mark on the Drug Price Amendment to Senator Kennedy | 09/13/1991 |
| Letter from Senator Edward M. Kennedy to Mr. Gerald J. Mossinghoff, Pharmaceutical Manufacturers Association President | 09/16/1991 |
| Letter from Senator Edward M. Kennedy to Colleagues | 09/30/1991 |
| Memorandum from Marsha Simon, Mark, and Ron on the Hearing on Drug Pricing and the Public Health to Senator Kennedy | 10/11/1991 |
| Public Health Clinic Prudent Pharmaceutical Purchasing Act, 1991, Hearing on S 1729 Before the S. Comm. on Labor & Human Resources, 102nd Cong, 1st Sess (1991) (Statement of Jose Camacho, Executive Director of the Texas Association of Community Health Centers on behalf of the National Association of Community Health Centers) | 10/16/1991 |
| Public Health Clinic Prudent Pharmaceutical Purchasing Act, 1991, Hearing on S 1729 Before the S. Comm. on Labor & Human Resources, 102nd Cong, 1st Sess (1991) (Statement of Leslie Tarr Laurie, Executive Director of Family Planning Council of Western Massachusetts) | 10/16/1991 |
| Public Health Clinic Prudent Pharmaceutical Purchasing Act, 1991, Hearing on S 1729 Before the S. Comm. on Labor & Human Resources, 102nd Cong, 1st Sess (1991) (Statement of Micheal A. Silver, M.D., Medical Director of The Providence Center for Counseling and Psychiatric Services) | 10/16/1991 |
| Public Health Clinic Prudent Pharmaceutical Purchasing Act, 1991, Hearing on S 1729 Before the S. Comm. on Labor & Human Resources, 102nd Cong, 1st Sess (1991) (Statement of Richard Samson, Director of Maryland Alcohol and Drug Abuse Administration) | 10/16/1991 |
| Public Health Clinic Prudent Pharmaceutical Purchasing Act, 1991, Hearing on S 1729 Before the S. Comm. on Labor & Human Resources, 102nd Cong, 1st Sess (1991) (Statement of Stephen W. Schondelmeyer, Pharm. D, Ph.D., Professor of Pharmaceutical Economics, College of Pharmacy, University of Minnesota; Director, PRIME Institute) | 10/16/1991 |
| Memorandum from Marsha Simon and Mark on the Meeting Tomorrow Afternoon with Searle's CEO, Sheldon Gilgore to Senator Kennedy | 11/05/1991 |
| Memorandum from Marsha Simon and Mark on the Discount Drug Bill, S. 1729 to Senator Kennedy | 11/13/1991 |
| Memorandum from Marsha Simon and Mark on the Discount Drug Bill to Senator Kennedy | 11/20/1991 |
| Memorandum from Marsha Simon and Mark on Senator Hatch's Offer on the Discount Drug Bill to Senator Kennedy | 11/22/1991 |
| Memorandum from Marsha Simon and Mark on S. 1729, Public Health Service (PHS) Clinic Discount Drug Bill to Senator Kennedy | 01/24/1992 |
| Memorandum from Marsha Simon and Mark on Drug Pricing Proposal of Senate Working Group to Senator Kennedy | 02/06/1992 |
| Hearings on HR 2890, HR 3405, and HR 5614 Before the Subcommittee on Health and the Environment of the House Committee on Energy and Commerce, 102nd Cong, 2nd Sess (1992) (subcommittee notice) | 07/24/1992 |
| HR Rep No. 102-384, pt 2, at 11 (1992). | 09/22/1992 |
| Memorandum from Marsha Simon on EMK's Role in Drug Pricing Legislation to Carey Parker | 10/21/1992 |

Note: All primary source documents are on file with the corresponding author. Although some of the documents in the primary source list were publicly available, they are listed here if they were annotated or have handwritten comments.

**Timeline of the 340B Program Legislative History**

1990: The Omnibus Budget Reconciliation Act of 1990 (OBRA 90) was passed, establishing the Medicaid drug rebate program. This led to unintended consequences, as pharmaceutical manufacturers began to raise prices for safety-net clinics to avoid giving Medicaid their "best price".

1990 - 1991: In the wake of the Medicaid drug rebate program, safety-net providers experienced significant cost increases. A report by the General Accounting Office (GAO/HRD-91-139) found that the VA health system's costs increased by an estimated $28 million.

July 1991: Senator Barbara Mikulski proposed an amendment (S. Amdt. 785) to the HUD-VA appropriations bill (H.R. 2519) to exempt VA prices from the Medicaid best price calculation. A staffer for Senator Kennedy worked with Senator Harkin to draft an amendment to an appropriations bill to address rising drug costs for Public Health Service (PHS) clinics. Representative Montgomery also introduces H.R. 2890, a bill aimed at protecting the VA from rising drug costs by linking it to the Medicaid program and exempting it from best price calculations.

September 16, 1991: Senator Kennedy sent a letter indicating that if a voluntary proposal from drug manufacturers was not presented, he would be forced to pursue legislation.

September 19, 1991: Senator Kennedy introduced S. 1729, the “Public Health Clinic Prudent Pharmaceutical Purchasing Act,” to establish a rebate program for PHS clinics.

September 24, 1991: Representative Wyden introduced H.R. 3405, which was the companion bill of S. 1729.

October 11, 1991: In a break from the pharmaceutical lobby, Pfizer sent a letter supporting legislation to provide drug rebates to PHS clinics.

October 16, 1991: A hearing was held on Senator Kennedy's bill, S. 1729.

October 28, 1991: The appropriations bill containing Senator Mikulski's amendment to protect the VA's drug pricing was passed.

November 6, 1991: Searle’s CEO, Sheldon Gilgore, asked for a meeting with Senator Kennedy to show support for discount prices for PHS clinics.

November 1991: After a markup in the Veterans Committee, H.R. 2890 is referred to the House Energy and Commerce Committee for further work.

January 1992: By this month, five additional pharmaceutical companies (Merck, Glaxo, Sytex, Astra, and Bristol Meyers Squibb) had proposed voluntary discount programs, showing a split within the industry.

February 1992: A bipartisan coalition of five senators, known as the "gang of five" (Cranston, Kennedy, Mikulski, Pryor, and Rockefeller), begins to form to work on a legislative proposal.

March 1992 (Spring): The "gang of five" starts to develop a proposal that would guarantee drug discounts for the VA and, importantly, allow Public Health Service (PHS) clinics to receive the same discounts.

May 18, 1992: Representative Montgomery introduced H.R. 5193, the “Veterans Health Care Act of 1992,” which later became the core 340B legislative framework.

June 1992 (Summer): The "gang of five" continues their work on the legislative proposal throughout the summer.

July 31, 1992: Representative Henry Waxman holds a hearing where public hospital representatives, including Dr. Ron Anderson of Parkland Memorial Hospital, argue that safety-net hospitals are being harmed by inflated drug prices and should be included in any discount program.

September 1992: The House Energy and Commerce Committee issues a report on its version of the bill, which now includes the PHS clinics. The report emphasizes the need to protect both the VA and federally funded clinics from manufacturer price increases.

October 1, 1992: The "gang of five" formally introduces their proposal as an amendment to S. 2575, a VA healthcare bill. Senator Cranston's initial amendment (S. Amdt. 3371) focused on securing discounts for the VA and DOD, and Senator Kennedy quickly added a co-sponsored amendment (S. Amdt. 3372) to include the PHS clinics.

October 1992: During Senate debate, the language developed by the "gang of five" is substituted into the House's Veteran Affairs bill (H.R. 5193).

November 4, 1992: The Veterans Health Care Act of 1992, containing the language that created the 340B program, was signed into law by President George H.W. Bush.
